# Machine Learning Models for the Identification of Prognostic and Predictive Cancer Biomarkers: A Systematic Review

**DOI:** 10.3390/ijms24097781

**Published:** 2023-04-24

**Authors:** Qasem Al-Tashi, Maliazurina B. Saad, Amgad Muneer, Rizwan Qureshi, Seyedali Mirjalili, Ajay Sheshadri, Xiuning Le, Natalie I. Vokes, Jianjun Zhang, Jia Wu

**Affiliations:** 1 Department of Imaging Physics, The University of Texas MD Anderson Cancer Center, Houston, TX 77030, USA; 2 Centre for Artificial Intelligence Research and Optimization, Torrens University Australia, Fortitude Valley, Brisbane, QLD 4006, Australia; 3 Yonsei Frontier Lab, Yonsei University, Seoul 03722, Republic of Korea; 4 University Research and Innovation Center, Obuda University, 1034 Budapest, Hungary; 5 Department of Pulmonary Medicine, The University of Texas MD Anderson Cancer Center, Houston, TX 77030, USA; 6 Department of Thoracic/Head and Neck Medical Oncology, The University of Texas MD Anderson Cancer Center, Houston, TX 77030, USA

**Keywords:** personalized medicine, biomarker discovery, predictive biomarker, prognostic biomarker, subgroup identification, machine learning, deep learning, feature selection

## Abstract

The identification of biomarkers plays a crucial role in personalized medicine, both in the clinical and research settings. However, the contrast between predictive and prognostic biomarkers can be challenging due to the overlap between the two. A prognostic biomarker predicts the future outcome of cancer, regardless of treatment, and a predictive biomarker predicts the effectiveness of a therapeutic intervention. Misclassifying a prognostic biomarker as predictive (or vice versa) can have serious financial and personal consequences for patients. To address this issue, various statistical and machine learning approaches have been developed. The aim of this study is to present an in-depth analysis of recent advancements, trends, challenges, and future prospects in biomarker identification. A systematic search was conducted using PubMed to identify relevant studies published between 2017 and 2023. The selected studies were analyzed to better understand the concept of biomarker identification, evaluate machine learning methods, assess the level of research activity, and highlight the application of these methods in cancer research and treatment. Furthermore, existing obstacles and concerns are discussed to identify prospective research areas. We believe that this review will serve as a valuable resource for researchers, providing insights into the methods and approaches used in biomarker discovery and identifying future research opportunities.

## 1. Introduction

Personalized medicine is rapidly becoming a reality in healthcare. The strategies for utilizing an individual’s distinct clinical, genomic, genetic, and environmental data to guide decisions about disease prevention, diagnosis, and treatment are evolving at an exponential rate [1]. Personalized medicine allows for therapies to be administered to the subsets of patients with the best responses based upon on their individual features, and furthermore, the use of personalized biomarkers can enable pharmaceutical firms to improve the likelihood of success in their clinical studies [2]. To identify the subset of patients who would benefit from a treatment, biomarkers are becoming more important in personalized medicine, whether for prognosis, prediction, or dosage selection. Furthermore, they may aid in the detection of therapeutic and unfavorable reactions, and thereby gauge effectiveness and safety prediction a priori. Thus, biomarkers are critical tools for selecting the appropriate patients for treatment with certain pharmaceuticals, as well as for enabling personalized medicine [3]. Biomarkers are considered molecular indicators of heightened benefit of—or toxicity caused by—a certain medicine [4]; moreover, they can be referred to as a measurement variable associated with disease outcome [5].

**Figure 1:**
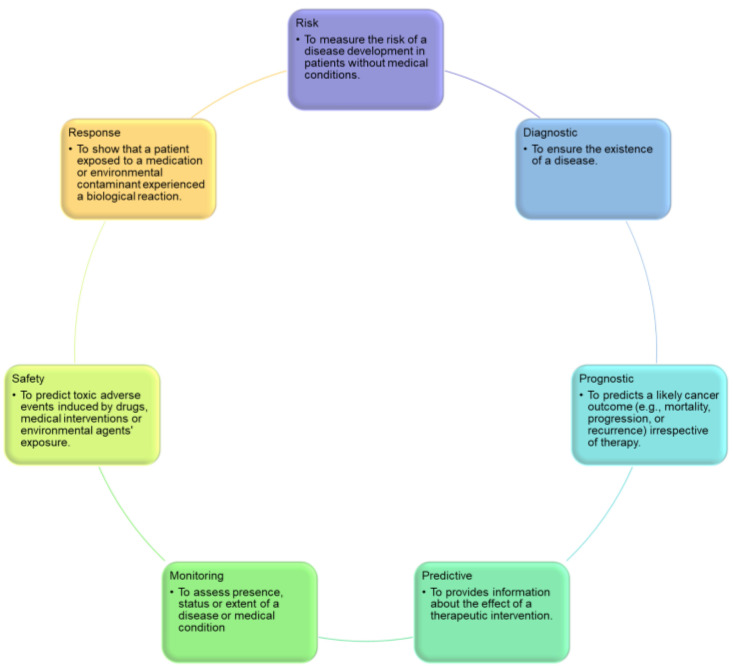
Categorization of biomarkers.

Biomarkers can be categorized into the following 7 types (Figure 1):

1. **Diagnostic biomarkers** predict the occurrence of an illness or classify people based on disease subtype. For example, individuals diagnosed with diffuse large B-cell lymphoma may be classified into subgroups using gene expression profiling of unique tumor cell signatures [6].
2. **Prognostic biomarkers** provide information regarding a potential cancer outcome, with or without therapy [5].
3. **Predictive biomarkers** indicate the likelihood of a patient’s response to a treatment plan and can be used to categorize patients as having a higher or lower chance of responding to a specific regimen, resulting in a gain in therapeutic precision [5].
4. **Monitoring biomarkers** are evaluated frequently over time to identify disease incidence, incidence or recurrence, disease progression, or other clinically relevant changes. For example, CA 125 is used to assess disease activity or burden in patients with ovarian cancer before and after surgery [7].
5. **Safety biomarkers** are evaluated prior to or following exposure to a therapeutic medication or environmental substance to determine the probability, frequency, and severity of toxicity as an adverse reaction. For example, serum creatinine in patients who are taking potentially nephrotoxic drugs is one of this biomarker type [8].
6. **Response biomarkers** demonstrate the physiological reaction of a patient to a medicinal drug or environmental contaminant. Plasma microRNAs, for instance, are used as a response biomarker for Hodgkin lymphoma [9].
7. **Risk biomarkers** indicate the likelihood that a person may develop an illness or health condition. They are particularly helpful for directing preventive actions in clinical practice. The BRCA1 and BRCA2 mutations, which evaluate the likelihood of breast carcinoma production, are two of the most recognized risk biomarkers, and BRCA carriers often undergo radical preventive measures to avoid the development of future cancers, such as elective mastectomy or salpingo-oophorectomy [7].

This systematic review is specifically centered on recognizing predictive and prognostic biomarkers using machine learning models because there is substantial uncertainty regarding the difference between the two [5]. Predictive and prognostic biomarkers are often mistaken for one another, and this can have negative medical, financial, and moral outcomes for patients and raises ethical concerns for clinicians and researchers. A prognostic biomarker mislabeled as predictive may result in overestimating treatment advantages for a segment of the population, resulting in prescription to certain individuals when it should be available to all. For example, though immunotherapy was initially indicated only in those with significant tumor expression of PD-L1, recent studies suggest that immunotherapy could be a viable treatment option for patients with low tumor expression of PD-L1 [10]. As a result, the drug’s price may increase because of its classification as a treatment for a definite subset of the patients, when in fact it should be available at scale to a broad range of patients. The opposite may be true in the event that a predictive biomarker is mislabeled as a prognostic factor. In this instance, the varying impact of the treatment on different subgroups of the inhabitants would be overlooked. The drug’s pricing may be affected because it is incorrectly assumed to have the same effect in all patients, whereas it should only be considered in select populations [11]. Such misbranding may lead certain drugs, which are effective in specific populations, to be removed from the market altogether due to poor financial performance.

In short, the line between predictive and prognostic indicators can be confusing. However, supposing we have knowledge of the underlying model that produces the data, it becomes much easier to demonstrate and agree on prognostic and predictive biomarkers. Mathematically, utilizing a linear model as a purely illustrative tool, the health outcome, *Y*, can be denoted as a function of the patient characteristics, *X*, and the treatment, *T*, as follows [11]:

(1) $$f\left(X,T\right)=\alpha_{1}X_{1}+\alpha_{2}X_{2}+(\beta_{2}X_{2}+\beta_{3}X_{3})T$$

The prognostic elements defined by coefficients$\;\alpha_{1}$ and $\alpha_{2}$ and coefficients $\beta_{2}$ and $\beta_{3}$ define the predictive elements. In the case of a continuous Y, the function f(X,T) can be used to represent the conditional mean E(Y|X,T), and if *Y* is binary, f(X,T) can be the logit of the conditional probability, i.e., logit[P(Y=1|X,T)]. $X_{1}$ has a direct influence on the outcome *Y* and is therefore considered a main effect and classified as a prognostic factor. $X_{3}$ is classified as a predictive factor as it only impacts *Y* through its interaction with the treatment variable, which is referred to as an interaction effect. Finally, $X_{2}$ is both prognostic and predictive, possessing both direct and interaction effects on *Y*.

Using the conventional notation [12], and assuming linear interactions, we can express the outcome function as [11].

(2) f(X,T)=h(X)+z(X)T

The functions h(.) and *z*(.) are arbitrary functions of the covariates. Two challenges arise in this context. The first challenge is to rank the variables in X based on their influence on *Y* through h(X), referred to as prognostic ranking. The second challenge is to rank the variables based on their influence on *Y* through z(X), referred to as predictive ranking. Assuming the binary treatment T∈(0, 1), a simple algebraic operation can be used to determine that z represents the change in the treatment-based outcome *Y*, which is commonly known as the treatment effect [11]:

(3) z(X)=f(X,1)−f(X,0)

The process of determining the treatment effect (by first estimating f(X,.)) inspires a range of techniques that make use of potential outcome modeling. This systematic review was carried out to carefully examine the existing approaches employed to identify prognostic and predictive biomarkers. Of note, this survey does not focus on discussing the technical difference between the two.

The rest of the paper is structured as follows: Section 2 outlines the method used to conduct the systematic review; Section 3 presents the results of the systematic study on the existing machine-learning-based approaches for prognostic and predictive biomarker identification, including information on machine learning methodology, the different approaches employed to identify prognostic and predictive biomarkers, the evaluation measures used to assess outcomes, the type of validation employed, main outcomes of the included studies, findings in relation to the research questions, and availability of source code; Section 4 highlights various applications of commonly used cancer biomarkers; Section 5 describes the subgroup identification methods; Section 6 discusses the results, ongoing problems, and future study directions; and Section 7 presents our conclusions.

## 2. Methods

### 2.1. Evidence Acquisition

#### 2.1.1. Aim

The primary goal of this systematic review was to evaluate and analyze recent research articles in the literature regarding the identification of prognostic and predictive biomarkers. In particular, we analyzed the studies that used machine learning to identify either of these biomarkers.

To this end, the following questions were established as our research focus:

- **RQ1**: What machine learning models are currently being utilized to identify prognostic and predictive biomarkers?
- **RQ2:** What kinds of model validation techniques have been utilized during the construction of machine learning models to identify biomarkers?
- **RQ3**: What metrics have been utilized to evaluate the efficacy of the machine learning models in detecting the biomarkers?
- **RQ4**: What are the key cancer applications used to validate the existing machine learning models?

#### 2.1.2. Search Strategy

A search of the literature was conducted using the National Library of Medicine’s PubMed database (https://pubmed.ncbi.nlm.nih.gov, accessed on 2 January 2023) to identify papers published between 1 January 2017 and 1 January 2023. The search was performed using a combination of keywords, including ((prognostic biomarker) AND (predictive biomarker)) AND (machine learning), and adhering to the Preferred Reporting Items for Systematic Reviews and Meta-Analyses guidelines [13]. Original research articles were thoroughly evaluated; review papers, abstracts, and reports from meetings were excluded.

#### 2.1.3. Study Selection Criteria

The criteria for selection serve to identify the best fitting research articles for the systematic review and eliminate ill-fitting ones. We used the following inclusion criteria to identify studies to include in this review:

1. The study had to be published between 1 January 2017 and 1 January 2023.
2. The study must be related to the use of machine learning models in the identification of prognostic and predictive biomarkers.
3. The study must include only cancer disease biomarkers (any type).
4. The study must have been published in a peer-reviewed journal.
5. The article must have a full-text version, and the most comprehensive version was included, if applicable.

The following criteria were used to exclude studies from this review:

1. The study was published before 1 January 2017 or after 1 January 2023.
2. The study was published in an informal location or unknown source, or the paper was irrelevant to the domain of machine learning for identifying prognostic and predictive biomarkers.
3. The study focused on biomarkers of non-cancer disease(s).
4. The study was published in a language besides English or the publication had already been selected for the study.
5. Reviews, systematic reviews, meta-analyses, and abstract publications were excluded.

### 2.2. Evidence Synthesis

**Figure 2:**
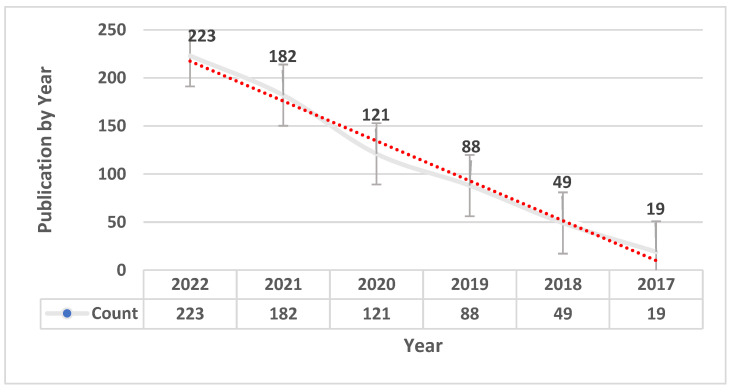
The trend of published number of studies on biomarker identification using machine learning.

**Figure 3:**
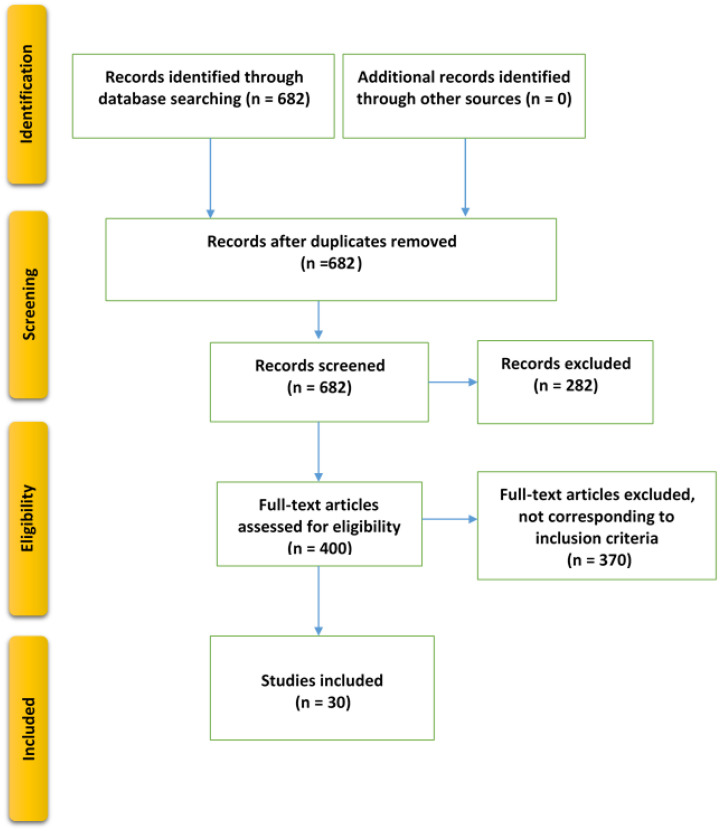
PRISMA flowchart [13].

Initially, 682 article references were obtained from the database search. The tendencies of prognostic and predictive biomarker discovery using machine learning models have increased from 2020 to 2022, as shown in Figure 2. Among the initial references, 282 articles were removed for being reviews, systematic reviews, meta-analyses, or abstracts or otherwise not meeting the inclusion criteria. Next, we obtained and evaluated the full text of the remaining 400 articles using several filtration steps and the inclusion criteria. First, 4 authors (Q.A-T., M.B.S., A.M., and R.Q.) screened the papers based on their title, and 87 papers were selected for the next filtration step. Second, the authors screened the abstracts, and 40 papers were selected for the final filtration criteria (full-text screening). Third, the full text of these 40 papers was then evaluated and critically analyzed, and 10 of them were excluded. Finally, 30 articles were deemed suitable for critical review and meta-analysis, as shown in Figure 3.

The following quality assessment guidelines aim to minimize bias and improve the transparency and repeatability of the systematic review process: focusing on identifying predictive and prognostic cancer biomarkers through the utilization of machine learning models, ensuring that the selected studies match the goal of the systematic review, checking if any performance metrics were used, assessing the reasonableness of the selected studies’ conclusions, and verifying that a legitimate data set was utilized.

## 3. Results

For the critical review and meta-analysis, 30 articles were analyzed. A metadata table (Table 1) was compiled to gather the most relevant information regarding the research questions described in Section 2.1.1 from the selected articles. The metadata table includes the reference of the publication, the year of publication, the publisher, the machine learning methodology applied, the objective of the article (i.e., prognostic or predictive modeling), the metrics used to evaluate performance, the validation model, the main results, key findings relevant to the research questions, and the source code/package. The metadata table is used to address the research questions (RQ1, RQ2, and RQ3) of the systematic review. The application section addresses RQ4 in Table 2. The data from the articles are extracted and analyzed systematically to address the four research questions, and conclusions are drawn based on the answers to these research questions. In sections below, we discuss each of the four research questions in detail. Finally, we evaluate the current state of the research and determine the direction for future development.

## 4. Application

**Figure 4:**
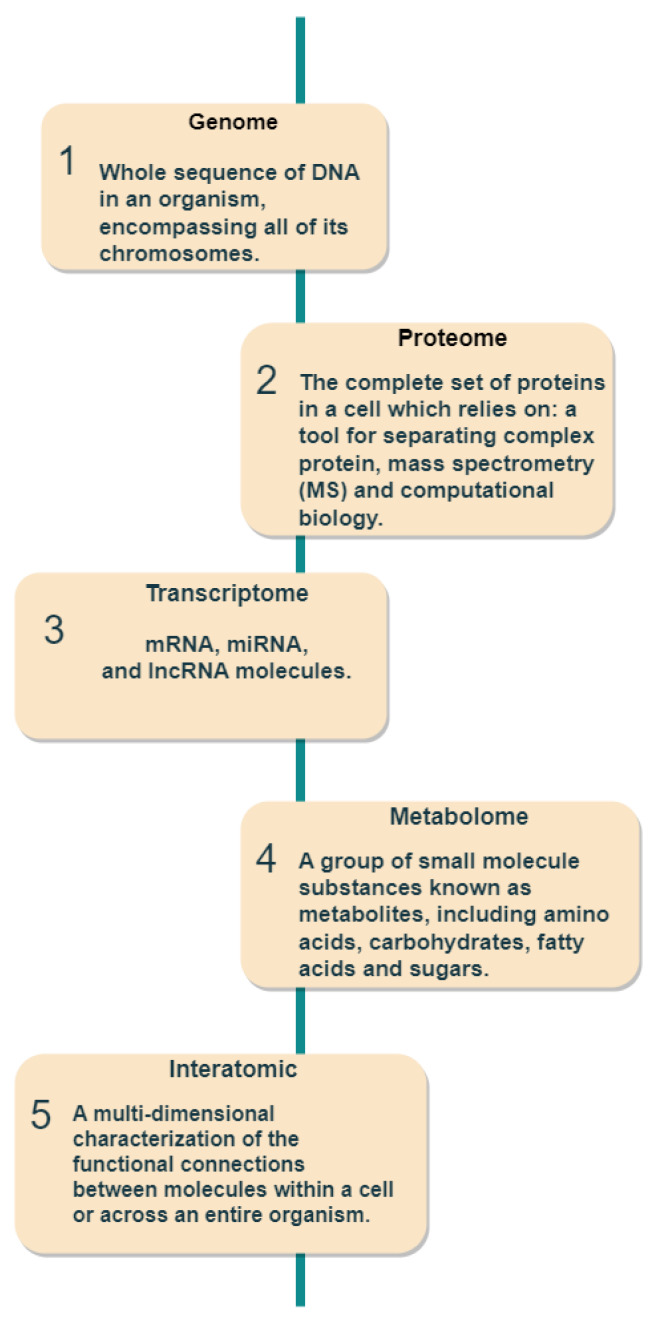
Omics data used for prognostic and predictive biomarker discovery.

This section describes the use of applications or datasets in the discovery of the two biomarkers (prognostic and predictive). A new age of omics in the discovery of biomarkers has begun as a result of several developments over the past few decades, including next-generation sequencing and microarray tools [43]. As can be seen in Figure 4, the omics data can be classified into five types as follows:

- Genome: medical genomics aims to detect genetic variations that correlate with illness, treatment efficacy, and patient prognosis [44].
- Proteome: detects changes in protein expression induced by a definite stimulus at a particular moment and identifies the configuration of protein networks at the cellular, organismal, or tissue level [45].
- Transcriptome: RNA serves as the intermediary among DNA and proteins, acting as the primary conduit for DNA-derived information [46]. The RNA-Seq method is used to analyze the transcripts or atomic dataset.
- Metabolome: Metabolomics is conducted at various levels of metabolites, and any relative imbalances or disruptions that are comparatively abnormal indicate the presence of illness [47].
- Interatomic: Protein-protein interactions are belonging to this type of omics data [48].

Finding disease biomarkers through the use of only one type of omics data can be difficult, which is why multi-omics, or the integration of multiple types of omics data, is necessary in the discovery process. Despite the advantages of having multi-omics to assess markers for disease diagnosis and progression, it is still a formidable challenge to pinpoint accurate biomarkers among the multitude of genes and variants. Table 2 shows a collection of cancer biomarkers for various types of cancer, according to selected research studies. The description of and link to each data source is also provided.

## 5. Subgroup Identification for Precision Medicine

**Figure 5:**
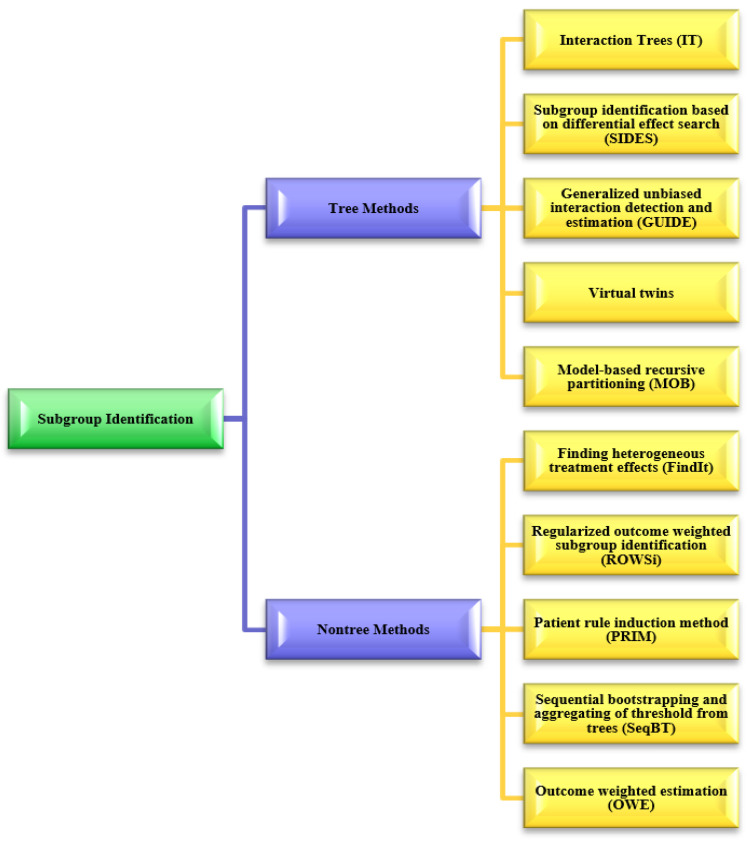
Subgroup tree- and non-tree-based methods for biomarker identification.

Because a treatment’s impact can differ greatly across the patient population, precision medicine aims to identify subgroups of patients whose average response to a treatment differs greatly from the population average [49]. We included subgroup identification methods in this systematic review because the methods used to identify subgroups are also used to identify predictive biomarkers. Statistically, a predictive marker has an impact on the treatment variable [50]. Figure 5 shows the most popular subgroup identification methods used in biomarker identification. These subgroup identification methods can be divided into two categories, namely: tree-based methods and non-tree-based methods [51]. Table 3 and Table 4 provide a detailed analysis of each method, including a description, objective functions, limitations, and the source codes links. A software package for several subgroup identification methods is available in BioPharmNet.

## 6. Discussion

This systematic review examines different methods for identifying prognostic and predictive biomarkers through the use of machine learning. Our systematic review focused on studies done within the past 5 years that relate to crucial research questions in this field. The purpose of the review is to highlight the biomarker identification methods that involve machine learning and deep learning, as their use is expected to be a prominent issue in the future because of the requirement for personalized treatment. This section will revisit and thoroughly discuss the research questions and then highlight the difficulties in biomarker discovery and provide future prospects and recommendations.

### 6.1. RQ1: What Machine Learning Models Are Currently Being Used to Identify Prognostic and Predictive Biomarkers?

**Figure 6:**
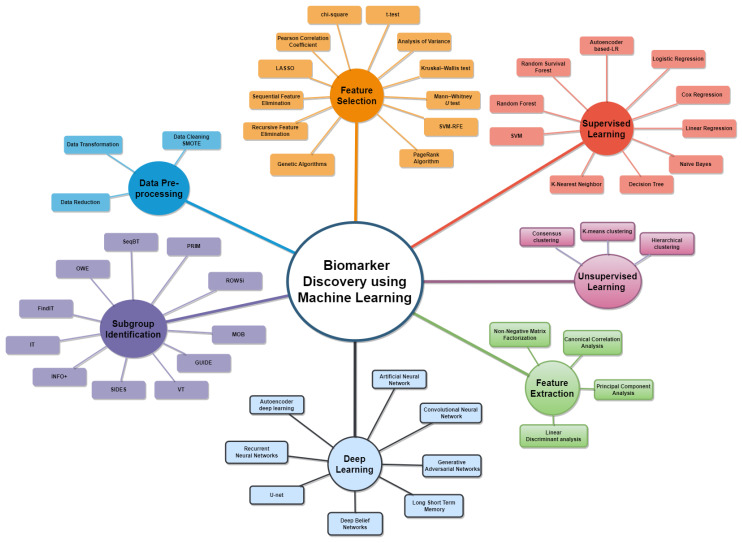
Map of machine learning approaches used for biomarker identification.

Figure 6 shows a map of the machine learning approaches used by the selected studies to identify biomarkers. Most of the selected studies first preprocessed the data, including cleaning the data to remove missing and noisy values, transforming the data into a specific range through normalization and selection techniques, and decreasing the dimensionality of a high-dimensional data set to a low-dimensional one. For example, in [20], the authors used SOMTE to handle the missing values. Second, most of the studies performed feature selection and extractions. Extraction of features entails decreasing the multi-omics data’s high-dimensional feature space to a lower dimensional one containing only vital information necessary for identifying biomarkers. Different techniques were used by the selected studies to extract the features, including principal component analysis as in [20] and non-negative matrix factorization as in [14]. Other similar approaches to extract features are canonical correlation analysis [66] and linear discriminant analysis [67]; however, such approaches work only with linear data.

When non-linear integration is necessary, as in the case of integrating interatomic data and gene expression, traditional techniques are not effective. In these situations, non-linear feature extraction techniques, including locally linear embedding [68] and kernel principal component analysis [69], as well as t-distributed stochastic neighbor embedding [70,71], are needed. Nevertheless, only a portion of features are obtained through feature extraction, and furthermore, relevant features must be selected through feature selection to identify biomarkers.

Feature selection is the process of identifying important and informative features by removing those that are duplicate or noisy [72]. Feature selection strategies can be wrapper, filter, and embedded types [73]. The filter one assesses the significance of characteristics based on their relationship to the outcome variable [74]. This can be accomplished using techniques such as Pearson correlation coefficient, analysis of variance, Spearman rank coefficient, *U* test, chi-square, Kruskal-Wallis test, and *t*-test, as in [15,24,30,34]. However, the filter method has a drawback in that it considers each feature independently, disregarding any complex relationships between features in omics data, which can lead to incorrect results. Furthermore, because the filter approach runs independently of the classifier, the chosen features perform poorly [75]. To overcome the drawbacks of filter approaches, wrapper methods were introduced. These methods iteratively select features and evaluate their performance through a classifier [76]. The process starts with no features, adds one at a time, and checks performance until the most relevant features are identified [77,78]. Features are selected using forward or backward feature selection. The common procedures used in wrapper methods include recursive-feature elimination, sequential-feature elimination, and genetic algorithms. As can be seen from the reviewed studies, the most common wrapper approaches used are recursive-feature elimination and forward selection as demonstrated in [11,18,29,30]. Two studies ([27,31]) used genetic algorithm to obtain prognostic and predictive biomarkers. However, the wrapper method can lead to overfitting.

To address this issue, the embedded scheme was introduced. The embedded technique merges both filter-wrapper methods, integrating the training procedure to the feature selection procedure to determine the optimal feature subset. One popular technique of feature selection in embedded methods is the least absolute shrinkage and selector operation (LASSO) method, which has been widely used to identify predictive and prognostic biomarkers as seen in [18,23,26,32,37,38]. Additionally, LASSSO was used to identify important features in most of the subgroup analyses. Nevertheless, LASSO has several drawbacks, such as being limited to selecting at most *n* variables and not being able to perform group selection. When there is a group of variables with a large number of pairwise correlations, LASSO often arbitrarily selects only one from the group, resulting in the disregard of important variables [79,80]. This drawback highlights the need for the development of better feature selection techniques to create more accurate prediction models to identify effective prognostic and predictive biomarkers, improve risk stratification, and facilitate personalized treatment.

In terms of modeling, machine learning enables machines to gain insight from errors, analyze data, recognize patterns, and produce informed judgments with minimal human involvement. Supervised, unsupervised, semi-supervised, and reinforcement learnings are the categories that machine learning domain includes [81]. However, in this systematic review the two most widely used methods were supervised and unsupervised learning.

Supervised one involves training the machine using labeled data that includes correct answers. The machine is then given test data to evaluate using any supervised algorithm, producing precise results [82]. Supervised learning can be categorized into either a classification or regression problem [83]. The result is a categorical or class variable in classification, while it is a real value in regression [84]. Based on the selected studies, the most commonly utilized machine learning models were Cox regression and variants for generating a signature gene that detects prognostic and predictive biomarkers (as seen in [14,19,21,24,25,26,29,32,33,34,35,37,38]). Besides, various supervised learning algorithms, such as DT, LR, NB, ANN, and RF were employed to forecast prognostic biomarkers and molecular subtypes. For instance, in [14], SVM with a linear kernel classifier was used to produce a risk score and to evaluate the effectiveness of non-negative matrix factorization subgroups. In [16], LR and autoencoder-based LR were used to screen pathogenic survival-related driver genes. In [17], RF and NB were used to identify important microRNAs as biomarker for predicting nilotinib response in chronic myeloid leukemia. Several studies in Table 1 used supervised machine learning models to predict prognostic and predictive biomarkers as well as classify the molecular subtypes of several cancers.

Unsupervised learning is a type of machine learning in which the computer analyzes uncategorized and unlabeled data [85]. The computer clusters the data into groups based on commonalities, differences, and variations without prior information. Unsupervised learning algorithms, such as K-means and hierarchical clustering, are instances of such methods. Among the selected papers, two studies used unsupervised learning: [14] used consensus clustering-based non-negative matrix factorization to identify molecular subtypes that are prognostic for survival and the studies [24,27,40], used K-means clustering to cluster the extracted representative features. Additionally, deep learning modeling was used in the selected studies to identify biomarkers. For instance, in [28], two convolutional neural networks were used to first outline cancerous tissue and then to stratify the patients into prognostic categories. Whereas in [34], a deep-learning-based method was used to extract prognostic biomarkers from computed tomography images of patients with ovarian cancer. In [41], GANs, which uses a candidate prognostic gene module, ranked genes according to multi-omics data in five different cancer types. Various tasks, such as classification, regression, and clustering, are accomplished using neural networks as explained in Table 1.

**Figure 7:**
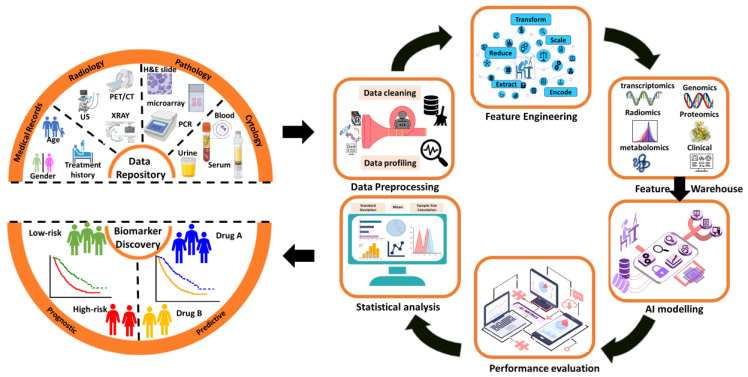
General machine learning framework for biomarker identification.

In summary, significant effort is being put into using computational and machine learning methods to find molecular signatures for disease prevention. Unlike traditional laboratory experiments, discovery of biomarkers using machine learning combines multiple data sources and biological knowledge to form a comprehensive understanding of the disease. With the growth of big data, the advancement of biomedical informatics and translational medicine is constantly advancing with new models and technologies to identify important markers related to health and disease stability. Network-based methods that rely on network topological and functional features, as well as machine learning algorithms such as SVM, RF, and clustering, are used to identify key factors affecting the stability and function of biological systems from large-scale expression data. It is worth noting that the identification of molecular biomarkers is not limited to single, static molecules. Because diseases can be dynamic and personalized, biomarkers at different time points or disease stages are increasingly recognized as markers for predicting abnormal interactions among biological components and making personalized clinical decisions. Finally, in the biomarker discovery field, diagnosing illnesses employs classification, predicting disease outcomes utilizes regression, and identifying biomarkers involves feature selection and extraction. The identified biomarkers are then inputted into machine learning or deep learning models, which categorize them into either (prognostic or predictive markers). To assess the survival analysis of prognostic markers, numerous techniques such as univariate and multivariate Cox are employed, and a risk score is computed to identify high-risk markers as prognostic markers. Relying on the results, treatments and drugs can be suggested. In contrast, identifying predictive markers is regarded as a gene prioritization problem, where new biomarkers can be discovered by studying known disease biomarkers. Various algorithms prioritizing genes and subgroup identification methods have been reviewed to identify predictive markers. Figure 7 shows a general machine learning framework in biomarker identification.

### 6.2. RQ2: Which Types of Model Validation Have Been Employed in the Development of Machine Learning Models for Biomarker Identification?

Model validation refers to the procedures and actions [86,87] that assess the accuracy of a machine learning model after it has been trained using a large data set, improving data quality and quantity, ensuring that the model is trustworthy before relying on its predictions. Model validation is particularly important in fields such as healthcare and biomarker identification, where any errors in prediction can have serious consequences. Some benefits of model validation include increased scalability and flexibility, reduced costs, improved model quality, the discovery of additional errors, and a reduction in overfitting and underfitting [86,87]. There are several techniques of model validation, including train/test split, K-fold cross-validation, leave-one-out cross-validation, and nested cross-validation. A detail of model validation can be found here [86]. As can be seen from Table 1, the percentage of selected studies that used different types of validation models: 50% of the selected studies used K-fold cross-validation, 17% used the train/test spilt method, and 7% used leave-one-out cross-validation. Moreover, 27% of the studies used training cohorts and independent validation cohorts. The small percentage of studies that used the latter are due to the fact that this method requires a significant amount of time to be fitted to the data set and can be computationally intensive if the model is complex.

K-fold cross-validation is used often because it avoids the overfitting problem encountered when cross-validation is not performed, especially for small data sets. This improvement, however, comes with a high cost. More computation power is required to find the best model when using K-fold cross-validation. It is also important to note that an independent testing set is recommended when evaluating the performance of the models for biomarker identifications. As shown in Table 1, there are a limited number of studies that validate their models with an independent testing set, and the majority used the same data cohorts for the final evolution of the models, leading to data leakage, which is one major limitation in the current studies.

### 6.3. RQ3: What Evaluation Measures Have Been Used to Assess the Performance of the Machine Learning Models in Identifying the Biomarkers?

The most commonly used metrics to assess the effectiveness of machine learning models in detecting cancer biomarkers are listed below:

- **The hazard ratio (HR):** A measure that compares the likelihood of an event occurring in a group receiving treatment to the likelihood of the same event happening in a group not receiving treatment, allowing researchers to determine if patients undergoing treatment experience an event faster (or slower) than those who are not [88].
- **The concordance index (C-index):** is widely used in survival analysis as a measure of discrimination [89] and is favored for its interpretability, as it is similar to classification accuracy and receiver operator characteristic area under the curve. Simply put, the C-index estimates the likelihood that, for a randomly selected pair of individuals, the predicted survival times are ordered in the same way as the actual survival times [90].
- **The log-rank test:** is a non-parametric statistical test used to compare the survival experiences between two groups and is commonly used in clinical trials to determine if one treatment leads to a longer survival time compared to another treatment [91]. Kaplan–Meier analysis and log-rank tests are usually used to evaluate the statistical significance between groups of patients [92].
- ***p*-values** are used to determine if an observed pattern is statistically significant (i.e., the *p*-value of a statistical test is low enough to reject the null hypothesis). The commonly accepted threshold for a low *p*-value is *p* < 0.05, which is roughly equivalent to the chance that the null hypothesis value (commonly assumed to be zero) falls within a 95% confidence interval [93].

Briefly, the log-rank test is frequently used to assess the hypothesis of similarity between the survival functions of two groups that have received different treatments in a randomized controlled trial. Additionally, trials often calculate HR to compare the risk of failure between the two groups. The HR is commonly estimated through the application of Cox proportional hazards model, and a 95% confidence interval is provided to reflect the accuracy of the estimated HR. For supervised learning and unsupervised learning, the evaluation measures differ based on whether the problem is related to regression, classification, or clustering. Some of these measures include accuracy, area under the curve, F1 score, precision, sensitivity, specificity and sensitivity, R^2^, and root-mean-square deviation. Complete details about these measures can be found in the literature [94,95,96]. Typically, evaluation metrics are divided into two main categories: discrimination and calibration metrics. Discrimination metrics evaluate the ability to accurately rank or distinguish between two classes. The most commonly used threshold-independent discriminative metric is the area under the receiver operating characteristic curve [97]. Another category of metrics assesses calibration, which measures the consistency between predicted probabilities and actual probabilities [98]. As can be seen from Table 1, the percentage of selected studies that used different types of evaluation measures: 67% of the selected studies used AUC and *p*-value, 47% used the HR, and 20% used C-Index. Moreover, 16% of the studies used the accuracy, 13% used log-rank and correlation coefficient. Other measures such as F1-score, precision, sensitivity, specificity, recall, root mean squared, mean absolute error, standard error, z-score, correlation coefficient and Pearson correlation have been used in few numbers of studies. Of note, each study could have several types of evaluation measures. For instance, the study in [34] utilized the following evaluation measures: C-Index, *p*-value, AUC, and HR.

### 6.4. RQ4: What Are the Key Cancer Applications Used to Validate the Existing Machine-Learning Models?

**Figure 8:**
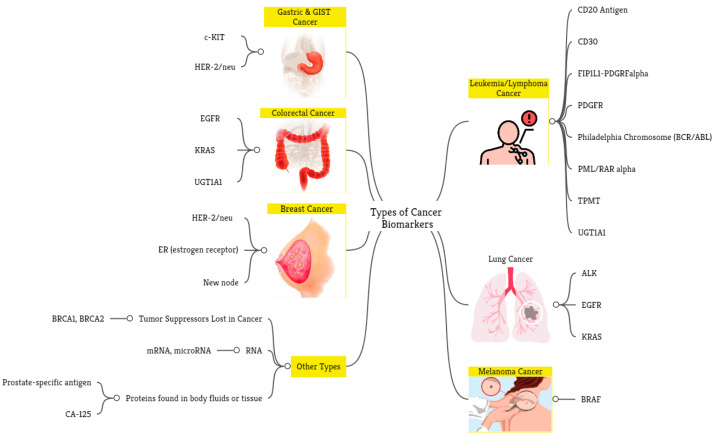
Types of cancer and biomarkers used by the selected studies in biomarker discovery.

In Section 4, a thorough examination of the clinical uses of cancer biomarkers was conducted. The analysis of the reviewed studies reveals that these biomarkers, also known as tumor markers, exist in various forms. They may include hormones and different groups of proteins, such as enzymes, glycoproteins, oncofetal antigens, and receptors. Moreover, cancer biomarkers also encompass genetic mutations, amplifications, translocations, and alterations in genetic signatures generated by microarrays. Figure 1 shows the categories of cancer biomarkers, and Figure 8 shows the biomarkers frequently used in the selected papers. As can be seen from the figure, different cancer types have been evaluated, including leukemia and breast, lung, gastric, colorectal, and skin cancers. Examples of biomarkers for each cancer type are provided in the figure as well. Of note, some studies used hematoxylin-and-eosin-stained slides and medical images beyond clinicogenomics to predict treatment outcomes [99,100,101,102].

### 6.5. Challenges in Biomarker Discovery

The reviewed studies demonstrate that biomarker discovery is a complex process that requires multiple steps from data processing to final model evaluation. Neglecting any of these steps can lead to false assumptions and incorrect predictions and identification of biomarkers. The challenges can be categorized into two major parts as follows:

First, in terms of cancer data sets, the existing data set is underpowered, meaning that there are more variables and attributes than the sample sizes. This problem creates the overfitting, making it challenging to identify biomarkers. To overcome this challenge, combining various types of data sets and using the integrated data set for biomarker discovery can be helpful. Additionally, biomarker discovery faces the heterogeneity of some molecular profiles, which can be either categorical or continuous and sometimes spread across multiple inputs. This heterogeneity makes biomarker discovery difficult. Another significant challenge is the data missingness, due to image noise, hybridization failures, and batch effects. As a result, a well-established imputation-missing value approach is necessary. The discovery of driver genes is another obstacle. While gene discovery can be performed using genomic data, it may not suffice for disease detection. As such, it is essential to integrate multi-omics data in order to precisely recognize the pivotal driver genes for disease prognosis and prediction.

Second, in terms of machine learning models, the current feature extraction methods are used for linear data; nevertheless, these methods prove to be ineffective when dealing with non-linear data, which require special extraction techniques. Feature selection methods used by the selected studies suffer from several drawbacks, such as the disregarding relationships between features in omics data in filter approaches, leading to inaccurate identification of biomarkers and an overfitting issue and complexity when applying wrapper approaches. Additionally, important variables are disregarded in embedded approaches when there are groups of variables with high pairwise correlations. There is an unmet need to involve a better feature selection technique to accurately identify effective prognostic and predictive biomarkers. Cox regression and its various forms have been extensively employed to develop signature genes that can detect prognostic and predictive biomarkers; however, this model suffers from several drawbacks. These include the model’s inability to account for non-linear relationships owing to its linear formula and the assumption that the effect of patient variables stays constant over time, which restricts the model’s ability to produce accurate predictions for patients at all follow-up time points. There is unmet need to develop an accurate model that incorporates both the non-linearity mechanism and robust feature selection approaches. Besides, there is still confusion regarding the differences between the prognostic and predictive biomarkers. Lastly, as can be seen in this study, most of the selected studies identified prognostic biomarkers, and there were fewer efforts to model predictive biomarkers.

### 6.6. Future Research Directions

According to current research studies, the following are potential future avenues of exploration in the area of biomarker discovery:

1. Developing feature selection approaches that overcome the limitations of existing approaches, e.g., swarm intelligence and meta-heuristic algorithms could help accurately identify prognostic and predictive biomarkers due to their robust performance in the feature selection field [103,104,105,106,107].
2. Developing or improving non-linear models that integrate deep learning algorithms, such as DeepSurv [108], for better signature gene identification with prognostic and predictive biomarkers.
3. Addressing the treatment effect that could be offered based on the biomarker’s identification, improving the current subgroup identification methods, and focusing more on the identification of predictive biomarkers.
4. Including more independent or external data cohorts to conduct a comprehensive investigation into the progression, diagnosis, and treatment of cancer.

### 6.7. Limitations of This Systematic Review

This systematic review of the literature has several limitations. One such limitation is that studies published outside of the time frame from 1 January 2017 to 1 January 2023 were excluded from the review. Additionally, only the PubMed database was used to search the published literature. This means that there may be important publications that were not analyzed and may have contributed to our findings. Additionally, to maintain the quality and reliability of the review, research papers that did not specifically focus on cancer biomarkers were excluded. Some of these non-cancer biomarkers were related to other applications and diseases, such as cardiovascular and metabolic diseases, and the identification of these biomarkers needs further investigation. Moreover, we limited our search to cancer predictive and prognostic biomarker identification. A future study could include other cancer biomarkers such as risk, monitoring, and safety biomarkers. We acknowledge that the use of filters in our systematic review may have influenced the conclusions drawn from the studies included. However, our exclusion criteria were carefully selected, and papers were carefully excluded following established criteria. Hence, the aim of this systematic review was to emphasize significant research in the field of identifying prognostic and predictive biomarkers through the use of machine learning models.

## 7. Conclusions

The objective of this systematic review of the literature was to give an overview of the current state of research in biomarker identification. The analysis was guided by four research questions. A preliminary search was carried out, and 682 studies were identified, but only 30 of them were considered relevant and selected for quality assessment after a thorough examination. The answers to the research questions helped to identify the challenges and problems in the field of biomarker identification using machine learning models. Additionally, this systematic review also investigated the use of subgroup models as a means of predictive biomarker identification and suggested future directions for the field, including the integration of modern feature selection techniques such as metaheuristic methods and the enhancement of non-linear models by incorporating deep learning algorithms. Recommendations were made to address the limitations found in the literature and to provide guidance for researchers and practitioners in the field. Additionally, the study acknowledges the limitations of the systematic review of the literature, including the exclusion of studies with poorly defined methodologies, short papers, and papers published in languages other than English. In conclusion, this systematic review of the literature aims to provide researchers with a comprehensive source of information about biomarker identification in one paper and serves as a starting point for future research in the field.

## Figures and Tables

**Table 1:** Meta-analysis and data extraction of the selected studies.

Refer	Year	Publisher	Machine Learning Methodology	Prognostic	Predictive	Measures	Validation	Results	Findings	Source Code
[14]	2022	Nature	An algorithm called (NTriPath) that utilizes machine learning was employed to discover a signature for gastric cancer. Unsupervised consensus clustering utilizing non-negative matrix factorization (NMF) was used to identify molecular subtypes that are prognostic for survival. Support vector machine (SVM) with linear kernel classifier was used to produce a risk score and to evaluate the effectiveness of NMF subgroups. Multivariable Cox proportional hazard analyses of overall survival were used within each genetic subgroup. Kaplan–Meier survival analysis was used to plot overall survival.	Yes	Yes	Hazard ratio (HR), *p*-value, risk score, log-rank, and area under the curve (AUC)	leave-one-out cross-validation and three independent test sets.	Using NTriPath, a gastric-cancer specific 32-gene signature was identified, and using NMF, 4 prognostic molecular subtypes were identified. The mean AUC for the classification was 0.981.	The 32-gene markers that were identified projected both overall survival (i.e., prognostic biomarkers) and response to treatments (i.e., predictive biomarkers). One group of patients obtained improved survival associated with adjuvant fluorouracil and platinum-based chemotherapy, and the other group had a worse response. The response to immune checkpoint inhibitors was linked to molecular classification.	NTriPath
[15]	2022	Springer	Perfusion and texture analyses were performed, and 160 parameters were extracted. First, a Mann–Whitney U test or t test was used to distinguish magnetic resonance imaging (MRI) parameters between pathologic biomarker groups. Second, a Kruskal–Wallis test and analysis of variance were used to compare MRI features among the four molecular subtypes. A post-hoc analysis was used to find different subgroups in case there were significant differences among subtypes in the above tests. Finally, five machine algorithms—logistic regression, decision tree, Naïve-Bayes (NB), random forest (RF), and artificial neural network (ANN)—were used to predict prognostic biomarkers and molecular subtypes.	Yes	No	AUC and *p*-value	Train-test split	Texture parameters were related with 6 parameters (*p* < 0.002). Perfusion parameters were associated with 4 parameters (*p* < 0.003). An RF model that combined texture and perfusion parameters produced an AUC of 0.75, which was the largest.	Integrating a radiomic model that incorporates angiogenesis characteristics and tumor heterogeneity holds promise in predicting prognostic factors for breast cancer. However, no treatment selection or predictive factors were identified in this study.	N/A
[16]	2022	Wiley	Machine learning analysis (logistic regression and autoencoder-based logistic regression) was used to screen pathogenic survival-related driver genes. Patient prognosis was analyzed by integrating copy number variation and gene expression data, and in silico analysis was presented to clinically assess data from the machine learning analysis.	Yes	No	AUC, F1 score, precision, and sensitivity	Five-fold cross-validation.	Three genes were identified as survival-related genes by machine learning and in silico experimental analysis.	The survival prediction approach provided information on patients and developing a therapeutic strategy for patients with colorectal cancer. No predictive biomarkers were reported.	N/A
[17]	2022	Nature	RF and NB machine-learning-based algorithms were used to identify important microRNAs as biomarkers for predicting the response of Nilotinib in chronic myeloid leukemia.	No	Yes	HR and receiver operator characteristics (ROC)	Ten-fold cross-validation	The combination of miR-145 and miR708 was an excellent predictor of nilotinib response in treatment-naive individuals, whereas miR-150 and miR-185 were significant classifiers at 1 month and 3 months after nilotinib therapy.	This study demonstrated that the integration of NL-CFC output into these panels enhanced their predictive ability. Therefore, this innovative predictive model may be adapted into a clinical prognostic tool.	N/A
[18]	2022	Nature	The authors attempted to predict an autoantibody-based biomarker panel for lung cancer using recursive-feature elimination with RF modelling and used least absolute shrinkage and selector operation (LASSO) regression with repeated 10-fold cross-validation.	No	Yes	AUC, sensitivity, specificity, ROC, and *p*-value	Ten-fold cross-validation	Strong expressers of an autoantibody-based biomarker profile had noticeably poor survivability, with an overall 5-year survival rate of 7.6%.	A profile of 13 predictive biomarkers outperformed the autoantibody biomarkers approaches adopted in solid malignancies for predicting survival in post-operative early-stage lung cancer.	N/A
[19]	2021	Frontiers	Univariate and multivariate Cox regression analyses were applied to evaluate the prognostic value of the autophagy-related long non-coding RNA (lncRNA) signature and to validate the association between the signature and survival of osteosarcoma patients in an independent cohort.	Yes	No	AUC, HR, z value, and *p*-value	Training and independent validation	Sixty-nine autophagy-related lncRNAs were identified, of which thirteen were significant predictors of the overall survival of patients with osteosarcoma.	Thirteen autophagy-related lncRNA prognostic biomarkers were identified in patients with osteosarcoma. No treatment selection had been performed.	N/A
[20]	2021	Nature	Gradient-boosted trees trained on histological grade 1 and grade 3 data. Several techniques were applied, including the detection of outliers using K-nearest neighbor, balancing the imbalanced dataset using the synthetic minority over-sampling technique, finding the best set of hyperparameters through a grid search on the training set samples and measuring the average performance across all cross-validation sets using the gain metric. To determine the most important features for classification, Shapley additive explanations was used. Finally, a principal component analysis was used.	Yes	No	Accuracy, gain, and Shapley additive explanations metrics	Ten-fold cross-validation	The identification of a 70-gene signature for determining clinical risk was demonstrated to be 90% accurate when used to evaluate samples with a known histological grade.	This model can classify high- and low-risk groups, without using clinical data, such as tumor size, tumor stage, or breast cancer subgroup. However, no experiments identified the responses to a specific treatment.	N/A
[21]	2021	Taylor & Francis	In this study, a multivariate analysis was employed to find the most significant prognostic proteins and calculate a risk score based on the expression of these proteins and the patients’ survival rate. Patients were divided into two groups: high risk or low risk, based on the median risk score. The correlation between the patient’s prognosis and the risk score was verified using survival curves and scatter diagrams. A heat map was created to visualize the expression of each candidate protein in the two groups. Univariate and multivariate Cox regression analyses were performed to determine the independent prognostic factors, which were used to build the IPRPs model. A cut-off value of 0.4 was used for the Pearson correlation analysis to determine the proteins associated with the IPRPs model. Results were considered significant if the *p*-value was less than 0.05.	Yes	Yes	AUC, log-rank test, and *p*-value	A validation cohort of 81 patients was used.	The prognosis-related protein model achieved diagnostic accuracy and had consistent predictive ability (AUC = 0.714). The results indicated that baseline SRC expression levels correlated with improved survival outcomes but predicted a worse response to immunotherapy.	This study highlighted the significance of the biomarker SRC for improved prognosis, and this newfound role has the potential to enhance predictive outcomes for patients receiving immunotherapy and aid in the selection of patients for future clinical treatment.	N/A
[22]	2021	Wiley	This study aimed to determine the diagnostic and prognostic capabilities of machine learning algorithms for suspected sepsis cases. The model was built on a clinical dataset of 1400 samples and uncommonly measured biomarkers. The machine learning analysis used three uncommonly used biomarkers (PCT, IL-6, and CRP) and data from electronic medical records (a patient’s age, sex, Glasgow coma scale, vital signs, and laboratory measures). RF was used to build the predictive models, and the data were divided in a 2:1 train-test split. Feature importance was completed using permutation-based tests, and five-fold cross-validation was used for the hyper-parameter tuning.	Yes	Yes	Area under the receiver operator curve (AUROC), area under the precision and recall curve (AUPR) and *p*-value	2:1 training: testing cohort ratio and 5-fold cross-validation.	For diagnostic performance, the AUROC was 0.83, and the AUPR was 0.61. For prognostic performance, the median length of stay was 3.2 days for 273 low-risk patients, 5.0 days for 164 moderate-risk patients, and 8.5 days for 30 high-risk patients (*p* = 0.0001).	A machine learning technique that incorporates fundamental clinical information and uncommonly measured biomarkers successfully diagnosed sepsis. As for release time, 30-day mortality and 30-day inpatient readmission, a higher score produced by the algorithm indicated fewer favorable outcomes.	N/A
[23]	2021	Public Library of Science	Several regression models were employed to examine the relationship between gene expression values and overall survival time. The regression techniques used included linear, ridge, LASSO, LASSO-Lars, elastic-net, RF, and K-nearest neighbors. The predicted overall survival times from the five sets of test data were combined and stratified using median cut-off, and HR, confidence interval (CI), and *p*-values were calculated. Hyperparameter optimization and regularization was performed using the grid search function.	Yes	No	HR, 95% CI, root mean squared error and mean absolute error.	Five-fold cross-validation	The expression pattern of prognostic genes was validated at the mRNA level, revealing differential expression between normal and PTC samples. Additionally, the HPA immunostaining results supported these observations.	The work elucidated the important prognostic biomarker genes in the apoptotic pathway whose aberrant expression relates to the progression and aggressiveness of PTC. Moreover, the proposed risk assessment models can aid in the efficient management of patients with PTC.	N/A
[24]	2021	MDPI	The autoencoder model was trained using a gradient descent algorithm. A univariate Cox regression analysis was performed. Then, the samples were grouped using K-means clustering, and the optimal number of clusters was established using the silhouette index and elbow techniques. Spectral clustering was applied for the SNF portion, and the best number of clusters was determined using the Eigen-gaps and rotation cost methods. The Wilcoxon rank-sum test was used to distinguish between the subgroups at high risk and low risk for recurrence in regard to differentially expressed genes, methylation-related genes, and miRNAs. The concordance index was calculated using the Cox-PH model. SVM classifier was built based on the labeled subgroup, which was made up of the top omics features selected through the Wilcoxon rank-sum test and clinical information.	Yes	No	C-index, log-rank, and *p*-values	Five-fold cross-validation	The multi-omics biomarker-based risk score was found to be a reliable predictor of prostate adenocarcinoma (PRAD) recurrence. Six overlapping omics biomarkers were selected for the multi-omics panel construction, includingTELO2, ZMYND19, miR-143, miR-378a, cg00687383 (MED4), and cg02318866 (JMJD6; METTL23). The results showed that the *p*-value was 5.33 × 10^−9^ and the C-index was 0.694.	This study contributes to a better understanding of the origin and underlying mechanisms of PRAD and offers patients and healthcare providers potential prognostic markers for therapeutic choices after surgical intervention. However, no predictive biomarkers were identified, and no treatment selection was suggested.	N/A
[25]	2021	Nature	An open-source deep learning algorithm was used to find tumor-infiltrating lymphocytes (TILs) in early-stage hematoxylin and eosin (H&E) slides of melanomas. The authors looked at the accuracy of automated digital TIL analysis (ADTA), based on current pathology standards, for the prediction of disease-specific survival (DSS). A cutoff value was established using an ROC and multivariable Cox proportional hazards were used for stratification.	Yes	No	HR, C-index, and *p*-value	Independent cohorts from 2 different institutions (total sample = 145 patients)	The results demonstrated that ADTA helped predict DSS (HR = 4.18, CI 1.51–11.58, *p* = 0.006). For inclusion in staging algorithms, ADTA offers an assessment of TILs and should be examined in larger research studies.	The study showed that digital pathology images can be analyzed to provide estimates of TILs that improve standard pathology assessments and have the potential to contribute meaningfully to clinical care. ADTA improved prognostic accuracy (*p* = 0.006).	QUIP QUPATH
[26]	2020	Wiley	In this study, two feature selection techniques, LASSO and SVM-recursive feature elimination, were used to identify potential lncRNAs for further analysis. The selected lncRNAs were then evaluated using univariate and multivariate Cox regression analyses to develop a seven-lncRNA signature for breast cancer prognosis. Overall survival was visualized using Kaplan–Meier analysis.	Yes	No	Coefficient, HR, standard error, Z score, and *p*-value	Training, validation, and external validation cohort	Seven lncRNA biomarkers have been identified, and the performance of this model was better relative to previous models.	The 7-lncRNA signature is a potential prognostic tool for predicting the overall survival rate of breast cancer patients. However, no predictive biomarkers were identified.	N/A
[27]	2020	Elsevier	A representative feature extraction was performed using an autoencoder neural network, followed by clustering using k-means. To construct a compact 5-gene prediction model, genetic algorithms were utilized. The effectiveness of the 5-gene class model was evaluated using logistic regression models.	Yes	Yes	AUC, chi-square test, and *p*-value	Training and external testing datasets	The study found 2 classes within the training cohort, with class 1 having a higher proportion of sepsis (21.8%) than class 2 (12.1%) with a significant difference (*p* < 0.01) as determined by the chi-square test. Five genes, *C14orf159*, *AKNA*, *PILRA*, *STOM*, and *USP4*, were identified using genetic algorithm. The performance of the 5-gene model was compared to that of other models in external validation cohorts, and it was found to be better at predicting mortality (AUC = 0.707, 95% CI: 0.664–0.750)	This research discovered two categories of sepsis that had different outcomes with regards to mortality and reaction to hydrocortisone treatment. Class 1 was associated with immunosuppression and had a higher mortality rate. Class 2 was relatively immune-competent. To determine class membership, a 5-gene class model was developed.	N/A
[28]	2020	Elsevier	This study used deep learning techniques to analyze scanned sections of H&E-stained tissue to create a biomarker for predicting patient outcomes following primary colorectal cancer surgery. This study utilized two convolutional neural networks to analyze patients with cancer. The first network outlined the cancerous tissue, and the second categorized patients into different prognostic groups. Univariable and multivariable analyses were used for risk stratification.	Yes	No	HR and *p*-value	Four different cohorts for training and tuning and one external test cohort	The validation cohort results showed that the HR for poor versus good prognosis was 3.84 (95% CI 2.72–5.43, *p* < 0.0001) in the primary analysis and 3.04 (95% CI 2.07–4.47, *p* < 0.0001) after adjusting for established prognostic markers such as pN stage, pT stage, lymphatic invasion, and venous vascular invasion.	A prognostic marker for patients with colorectal cancer was developed by using deep learning technology to analyze H&E-stained tissue sections that were digitally scanned. No treatment selection was made in this study.	N/A
[29]	2020	BioMed Central	The study used various machine learning techniques to identify 24 pairs of alternative splicing events (ASEs) related to lung adenocarcinoma (LUAD) and their impact on splicing. Additionally, an RF classifier was developed using 12 ASEs to predict lymph node metastasis (LNM) in LUAD patients. A 16-ASE-based prognostic model was established to predict overall survival in LUAD patients using Cox regression analysis, random survival forest, and forward selection method. Bioinformatics was used to examine the underlying mechanisms and associated upstream splicing factors. The results were confirmed by the Boruta algorithm, which also indicated the importance and selection of features.	Yes	Yes	Correlation coefficient and AUC	Five-fold cross-validation	The 12-ASE–based classifier for LNM demonstrated good accuracy and precision in a cross-validation study, with AUROC scores of more than 0.7 in all evaluations.	Alternative splicing AS may play a significant role in cancer progression. Most biomarkers identified in this study display AP, AT, and ES splicing patterns, which suggest they play a key role in the initiation and development of LUAD. However, there were no additional AS data available for validation. Second, the exact molecular mechanisms of these biomarkers are still unknown due to a lack of in vitro or in vivo experiments.	N/A
[30]	2020	Ivyspring International Publisher	Initially, the seed genes associated with survival were picked using the RF survival model. Then, the forward selection model, with the help of clinical RNA sequencing data, was used to determine the crucial genes among the seed genes. Then, a survival risk scoring system was established using these key genes in three patient data sets (cohort II, GSE72094, and GSE11969). Lastly, bioinformatics techniques such as pathway analysis, heatmap, and protein-gene interaction networks were applied to the seed genes and key genes.	Yes	No	HR, C-index, and *p*-value	Training and three independent validation cohorts.	Sixteen genes were found to predict the prognosis of LUAD patients with good precision in cohort II (HR = 3.80, *p* = 1.63 × 10^−6^, C-index = 0.656) and were further confirmed in the GSE72094 (HR = 4.12, *p* = 1.34 × 10^−10^, C-index = 0.672) and GSE11969 (HR = 3.87, *p* = 6.81 × 10^−7^, C-index = 0.670) cohorts.	A 16-gene prognostic marker for LUAD could be a useful tool for precise identification of cancer biomarkers. Nonetheless, no biomarkers with predictive capabilities have been discovered.	N/A
[31]	2020	Oxford University Press	An adaptive genetic algorithm called GARBO that operates across multiple islands has been developed to improve both accuracy and set size in omics-driven biomarker discovery challenges. The algorithm uses RF methods to assess classification accuracy and analysis of variance to produce a univariate feature ranking that informs genetic operations.	Yes	Yes	Accuracy and stability score, F1-score, Dice coefficient, and trade-off between accuracy and selected biomarkers.	Three-fold cross-validation for fitness function and ten-fold balanced and stratified cross-validation and independent test	GARBO is effective in selecting the optimal Pareto-based preferences. When dealing with omics data types that have large feature sets, such as mRNA, copy number variations (CNV), and DNA methylation. Other selection methods are more suitable for finding better trade-offs when working with DNA mutations and microRNA expression data.	The GARBO algorithm was applied to two biomarker discovery problems: cancer patient stratification and predicting drug sensitivity. It led to the identification of biomarker panels that were both clinically and biologically relevant and, in most instances, showed superior performance in terms of classification accuracy and set size compared to panels discovered using other methods.	GARBO
[32]	2020	Portland Press, Ltd.	A methylation prediction model was established in the training set by employing the “cv.glmnet” function within the “glmnet” package to determine the LASSO rank, and subsequently utilizing the “glmnet” by apply the Cox multivariate regression to compute LASSO. The model with the finest performance and the fewest number of independent variables was chosen according to the highest value of lambda. The samples were segregated into high- and low-risk categories, and the methylation prognostic prediction model was utilized to compare their survival rates. A significance level of *p* < 0.05 is used as the midpoint, and the predictive efficiency was assessed utilizing AUC.	Yes	No	Correlation coefficient, AUC, and *p*-values.	Train-test split	The following 10 genes were identified: *XIST*, *CCDC8*, *KRTCAP3*, *SMIM3*, *DCAF4L2*, *ZNF471*, *ALDOC*, *LGALS12*, *VEGFA*, and *AQP1*. The correlation coefficients ranged from −0.681 to −0.875 and showed a statistically significant difference (*p* < 0.05). The AUC values of the model in this study were 0.794, 0.752, and 0.731 for the 1-, 3-, and 5-year survival rates, respectively.	This study used machine learning to establish a multivariate methylation prediction model and integrate it with clinical information. Ten prognostic biomarkers were identified. However, no external data were used for validation, and no treatment selection was suggested.	N/A
[33]	2020	EPUB	A genome-wide DNA methylation analysis was performed on 299 colon cancer adenocarcinoma (COAD) samples and 38 normal-tissue samples from the TCGA. Using a training cohort, the researchers used conditional screening and machine learning to find one hypomethylated and nine hypermethylated CpG sites with substantial differential methylation. These locations were then utilized to develop a COAD diagnostic model as prospective diagnostic biomarkers.	Yes	Yes	AUC, *p*-value, and R-value (Correlation)	Independent validation cohort	The model accurately discriminated colon cancer from nine other cancer types, including breast cancer and liver cancer (with an error rate of 0.05), as well as from healthy tissues in the training cohort (AUC = 1). To validate the diagnosis model, a TCGA validation cohort (AUC = 1) and five independent cohorts from the Gene Expression Omnibus (AUC = 0.951) were used. The researchers created a predictive model based on six CpG sites using a Cox regression analysis and validated the model using data from the validation cohort.	The DNA methylation patterns of the genome’s CpG sites were employed to identify biomarkers and create machine learning models for COAD diagnosis and prognosis. The model accurately identified COAD from among samples of normal tissue and nine types of cancerous tissues. They also identified six CpG sites as potential prognostic biomarker and showed that the model could predict the prognosis of patients independently of important clinicopathological characteristics.	N/A
[34]	2019	Elsevier	A deep learning network was proposed to identify the inherent qualities of high-grade serous ovarian cancer (HGSOC) from preoperative computed tomography (CT) scans. This DL network was unsupervised learning using only the CT scans of HGSOC without any additional follow-up information. The feature-learning aspect was a convolutional autoencoder structure that transformed ovarian cancer into a 16-dimensional deep learning feature. The encoder and decoder network used skip connections. The recurrence analysis involved a multivariate Cox proportional hazard regression that used the DL feature to make predictions about recurrence.	Yes	No	C-Index, *p*-value, AUC, and HR	Two training (feature-learning and primary cohorts) and two validation cohorts were used	In the two validation cohorts, the model’s C-Index was 0.713 and 0.694. The Kaplan–Meier differentiated high and low recurrence risk (*p* values of 0.0038 and 0.0164, respectively). The 3-year recurrence prediction was also validated with AUCs of 0.772 and 0.825, respectively.	The deep-learning-based model offers a unique prognostic analysis tool that can determine prognostic biomarkers from CT data without the need for additional follow-up information. However, no treatment selection was performed in this study.	N/A
[35]	2019	American Association for Cancer Research	Seven classifiers were used to obtain prognostic insights from 32 parameters. These classifiers included gradient-boosting machine (GBM), SVM, RF, conditional RF (CRF), NB, neural network, and elastic net. The correlation between blood markers was determined using the Spearman rank coefficient. The difference in cancer recurrence was evaluated using univariate Cox proportional hazards models.	Yes	No	Accuracy, AUC, *p*-value, and HR	Train-test split	The RF classifier achieved the highest accuracy and AUC for separating epithelial ovarian cancer (EOC) from benign ovarian tumors with values of 92.4% and 0.968, respectively. The highest accuracy and AUC for RF in predicting clinical stages were 69.0% and 0.760, respectively.	This study’s supervised machine learning approach showed the correlation between preoperative blood markers and key features of EOC, which could be utilized for patient stratification. No treatment selection was performed.	CCR2019
[36]	2019	Nature	The objective of this research was to examine the ability of commonly used machine learning algorithms, such as DT, NB, RF, SVM, and ANN, to forecast prognostic markers and molecular subtypes of breast cancer through the analysis of perfusion features using CT. The five machine learning models were employed to analyze the 18 CT parameters of cancers to predict factors such as lymph node status, tumor grade and size, hormone receptor status, HER2 status, Ki67 expression, and molecular subtypes.	Yes	No	Accuracy and AUC	Train-test split	The RF model showed a 13% improvement in accuracy and a 0.17 increase in AUC. The most crucial CT parameters in the RF model for prediction were identified as peak enhancement intensity in Hounsfield units, time to reach peak, blood volume permeability in mL/100 g, and tumor perfusion in mL/min per 100 mL.	Applying machine learning techniques to radiogenomics through low-dose perfusion breast CT scans is an effective method for identifying prognostic biomarkers and molecular subtypes of invasive breast cancer. The combination of advanced CT technology and a robust statistical model makes radiogenomics of breast cancer a promising approach that can aid in risk categorization. However, no predictive biomarkers for treatment selection were used.	N/A
[37]	2019	MDPI	This study applies machine learning to develop a prognostic pseudogene signature for osteosarcoma. The authors screened pseudogenes that were associated with survival and used Cox regression analyses (univariate, LASSO, and multivariate) to construct a signature model. The signature’s predictive abilities were evaluated across various subgroups, and its potential biological functions were explored through co-expression analysis.	Yes	No	AUC, *p*-value, HR, and Pearson correlation	Ten-fold cross-validation	A signature composed of four pseudogenes (RPL11-551L14.1, HR = 0.65, 95% CI 0.44–0.95; RPL7AP28, HR = 0.32, 95% CI 0.14–0.76; RP4-706A16.3, HR = 1.89, 95% CI 1.35–2.65; and RP11-326A19.5, HR: 0.52, 95% CI: 0.37–0.74) effectively distinguished patients based on their risk level with a high accuracy in prognosis prediction (AUC = 0.878).	Four pseudogene prognostic biomarkers for osteosarcoma were identified. The four pseudogenes were found to control immunological, DNA/RNA editing, and the malignant phenotype by co-expression analyses. No treatment selection was suggested.	N/A
[11]	2018	Oxford University Press	An information-theoretic approach was employed that presents a mathematical framework for quantifying and discussing the individual predictive and prognostic strengths. An RF model was used to determine the prognostic score for each biomarker and virtual-twins counterfactual modeling was used to calculate the predictive score. A greedy forward-selection procedure was used to generate predictive biomarker rankings (INFO+) by estimating conditional mutual information values.	Yes	Yes	*p*-value and HR	Ten-fold cross-validation	INFO+ effectively captured higher-order interactions and separated the predictive and prognostic information of each biomarker more effectively, resulting in improved TPR and FNRProg performance.	The authors presented a visual illustration, the PP-graph, which embodies both the predictive and prognostic strengths of a group of biomarkers. This approach can be one of the subgroup identification methods aiming to differentiate between prognostic and predictive biomarkers.	INFO+
[38]	2018	Wiley	A machine learning method based on RF was applied to mRNA expression data of gastric carcinoma (GC), which consisted of 408 GC tissue samples and 36 adjacent non-tumor GC tissue samples collected from 350 patients. The LASSO Cox regression model was employed to identify the lncRNA signatures in the 36 adjacent non-tumor GC tissues.	Yes	No	AUC, Kaplan–Meier, *p*-value, and HR	Ten-fold cross-validation	Of the 6422 lncRNAs found to have different expression levels between tumor and normal tissues, a univariate Cox analysis identified 255 as prognostic lncRNAs.	The aim of this study was to identify and assess a prognostic signature of lncRNAs in patients with GC. No treatment or predictive biomarkers were identified.	N/A
[39]	2018	Nature	This study used tissue phenomics methodology, which encompasses a discovery process from development and image analysis to data mining, culminating in the final interpretation and validation of findings. This process is not linear and allows for backward steps and iterative optimization across multiple sub-processes. Specifically, the authors utilized automatic methods to identify tissue-based biomarkers with significant prognostic value for patients with low- to intermediate-risk prostate cancer (Gleason scores 6–7b) after undergoing radical prostatectomy.	Yes	No	Accuracy, *p*-value	Leave-one-out cross-validation	The phenotypes were associated with the presence of CD8 (+) and CD68 (+) cells in the microenvironment of cancerous glands, along with the local micro-vascularization. Predictive models based on these phenotypes achieved accuracy rates of up to 83% and 88%, respectively, for predicting tumor progression.	The outcomes of this paper have the possibility to be used in the future for prognostic testing for patients with prostate cancer and demonstrated the feasibility of the tissue phenomics methodology. However, no treatment selection was reported in this study.	N/A
[40]	2018	Nature	A network-based deep learning technique (G2Vec, which is a modified CBOW-based neural network) was employed to recognize both gene modules and prognostic biomarkers. The proposed method for gene selection involved: producing distributed gene illustrations, identifying L-groups using K-means clustering, and calculating gene scores. Prognosis was then projected over 10-fold cross-validation utilizing RF classifier.	Yes	No	AUC	Ten-fold cross-validation	G2Vec was found to outperform other methods (0.009–0.049 AUC) in utmost cancer forms, except for BRCA, for which G2Vec was not the best marker. AUROC values utilizing the FI network were a little better than those obtained from other networks, but the differences were not statistically significant across all cancer types.	The findings showed that G2Vec improved the accuracy of predicting patient outcomes compared to existing gene selection techniques. Moreover, G2Vec was able to recognize prognostic biomarkers related to hepatocellular carcinoma. No treatment or predictive biomarkers were identified.	G2Vec
[41]	2018	MDPI	The authors employed a graph-learning model based on generative adversarial networks that utilized multi-omics data, such as CNV, mRNA, DNA methylation, and SNP data, to rank genes within a candidate prognostic gene module for five different cancer types. Additionally, the PageRank algorithm was used for feature selection.	Yes	No	AUC and *p*-value	Ten-fold cross-validation	This method was able to identify genes associated with the development of cancer. The genes identified from various omics data sources showed limited overlap, leading to improved prediction accuracy when incorporating multi-omics data.	The proposed model was able to discover prognostic biomarkers, but no predictive biomarkers were identified.	N/A
[42]	2017	Nature	Researchers utilized two generalized linear models, 14 VF and 14 GT, both employing elastic net regularization, to analyze digital H&E images of clear cell renal cell carcinoma (ccRCC) tumors. The models were trained by transferring the outlines of endothelial cells from immunohistochemistry to H&E-stained images. By classifying ECs in ccRCC, the researchers were able to create vascular architecture maps, which were then used to identify biomarkers related to vascular morphometric features.	Yes	Yes	AUC, *p*-value, and C-index	Ten-fold cross-validation	A set of 9 vascular features was discovered and studied in relation to a 14-gene expression signature identified through correlation analysis and information gain analysis. The *p*-value was deemed significant at 0.036, and while the HR was only 1.65, the classification AUC was high at 0.96.	A model was developed using morphology-based gene expression profiles from vascular architecture, which was analyzed through digital image analysis and focused machine learning. The model is based on 14 genes and showed HRs of 2.4 and 3.33 for 14 vascular features and 14 GT, respectively. This approach has the potential to improve the identification of biomarkers using a unique morphogenetic methodology.	N/A

**Table 2:** Application of cancer biomarkers used by the selected research studies.

Refer	Cancer Type	Description	Data Source
[14]	Gastric adenocarcinoma	The research involved 612 patients with gastric adenocarcinoma who underwent surgery at Yonsei University from 1999 to 2010, 28 patients treated at Seoul St. Mary’s Hospital from 2018 to 2020, and 17 patients from Yonsei between 2014 and 2017. Additionally, the study incorporated cohorts from The Cancer Genome Atlas Project (TCGA), the Asian Cancer Research Group (ACRG), as well as the Sohn and Kim cohorts.	Gene expression profiles can be accessed from here and here. RNA-sequencing data, ACRG data, Sohn et al. cohort and Kim et al. cohort.
[15]	MRI (radiomic breast cancer)	Between May 2017 and July 2019, this study included 288 individuals who underwent breast magnetic resonance imaging (MRI) at 3 T before therapy, and a total of 291 lesions were detected in these patients.	N/A
[16]	Colon cancer	The gene expression, clinical, and CNV data for RNA-seq were obtained from TCGA (COAD).	TCGA-COAD
[17]	Chronic myeloid leukemia	Peripheral blood samples are collected from 62 patients who were recently diagnosed with chronic myeloid leukemia (CML), were enrolled in the Canadian sub-group of the phase IIIb clinical trial ENESTxtnd and were treated with heparin.	ENESTxtnd
[18]	Lung cancer	Pre-operative serum samples from 157 research participants (non-small cell lung cancer stage I–IIIa) were collected from a sizable database of trial patients and used in the proteomics analysis. Based on the standard deviations of each protein in the immunome array, a sample size calculation was made to obtain a power of 95%.	N/A
[19]	Bone cancer (osteosarcoma)	To investigate the gene expression and clinical information of 82 osteosarcoma samples with survival data, the researchers collected data from the Therapeutically Applicable Research to Generate Effective Treatments (TARGET) and Gene Expression Omnibus (GEO) databases. An autophagy gene list was obtained from the Human Autophagy Database, and the autophagy-related gene expression matrix was extracted using the TARGET and GEO databases.	TARGET GEO
[20]	Breast cancer	The researchers collected gene expression data from 33 datasets related to breast cancer, comprising a total of 5031 tumor samples and 70 normal samples. The datasets also included the clinical characteristics of the samples.	GEO
[21]	Bladder cancer	A total of 81 patients with bladder cancer who received intravesical chemotherapy treatments between August 2018 and June 2020. The pathology reports or EMRs supplied clinicopathological information. Samples of both bladder cancer and normal bladder tissue are obtained through surgery and were then handled and put in storage at the Fudan University Shanghai Cancer Center tissue bank.	N/A
[22]	Sepsis	A group of rare biomarkers and clinical information were evaluated in a 2-center study that prospectively collected samples from 1400 adult patients who presented with suspected sepsis in emergency departments. The data and specimens were collected during the period between February 2018 and September 2019.	N/A
[23]	Thyroid cancer	Quantile-normalized RNAseq expression levels for 573 patients with thyroid carcinoma were collected from TCGA in the original dataset. Out of these samples, 505 were analyzed.	N/A
[24]	Prostate cancer	This study used 494 patients with PRAD from TCGA portal, including 60 recurrent cases.	Genomic Data Commons (GDC)
[25]	Melanoma cancer	The training group’s images comprised 80 patients who were diagnosed with primary melanoma tumors that could be surgically removed between 2000 and 2014. The validation set was composed of 145 patients.	N/A
[26]	Breast cancer (BRCA)	The training dataset comprised 973 breast cancer cases, with 150 of them having triple-negative breast cancer (TNBC) and 823 being non-triple-negative breast cancer (non-TNBC) samples. The external validation cohort’s expression profile matrix and the patients’ clinical details from the GSE96058 dataset were obtained from the GEO database.	TCGA BRCA and GEO GSE96058 data sets.
[27]	Sepsis	The researchers searched the GEO and Array Express databases from their inception until April 2020 to identify datasets that included whole-blood gene expression profiling in adult patients with sepsis. A total of 12 datasets met the inclusion criteria.	GEO and Array Express
[28]	Imaging (colorectal cancer) histological sections stained with H&E	The training data were more than 12 million image tiles from four cohorts of patients with either a favorable or unfavorable disease outcome. To evaluate the prognostic marker, the researchers analyzed slides from a total of 920 patients in the U.K. and independently validated the findings in 1122 patients from Norway who were treated with a single agent capecitabine. All patients included in the study had resectable tumors and formalin-fixed, paraffin-embedded tumor tissue available for analysis. The primary outcome of interest was cancer-specific survival. A predefined protocol was used for both cohorts.	N/A
[29]	Lung adenocarcinoma	The RNA sequencing data and alternative splicing data were obtained from the TCGA database and TCGA SpliceSeq database, respectively.	TCGA TCGA SpliceSeq
[30]	Lung adenocarcinoma	The RNA sequencing data and clinical information for LUAD from TCGA were separated into two groups: TCGA cohort I with 338 samples and TCGA cohort II with 168 samples. The first cohort was used to build the model, while the second cohort and data from 2 other cohorts (GSE72094 and GSE11969) obtained from the GEO were used for validation.	TCGA GSE72094 GSE11969
[31]	Breast cancer	Omics data from three different sources—TCGA, The Cancer Cell Line Encyclopedia (CCLE), and Genomics of Drug Sensitivity in Cancer (GDSC)—were used to select sets of biomarkers for stratifying cancer patients and classifying drug-resistant or -sensitive cell lines. The effectiveness of this approach was evaluated by testing it on a total of 18 different omics datasets, which included 6 drugs for 3 different types of omics data, as well as 5 datasets for classifying breast cancer subtypes.	TCGA CCLE GDSC
[32]	Kidney renal clear cell carcinoma	The researchers obtained the methylation data, clinical data, and RNA-seq expression of KIRC using the TCGA website. The clinical data included information such as survival status, age, follow-up time, gender, and tumor stage, which were collected and analyzed. After matching the methylation data, clinical data, and gene expression value, a full amount of 317 tumor samples were selected for the study. Only samples with a minimum survival time of 30 days were included in the survival analysis, resulting in a total of 294 tumor samples.	TCGA
[33]	Colon cancer	The methylation levels of 10 types of tissue samples, including tumors and normal tissues, were obtained from TCGA. Five DNA Methylation arrays were included as independent cohorts from the GEO.	TCGA GEO
[34]	Ovarian cancer	In this study, 245 patients with HGSOC were included, consisting of a feature-learning cohort (*n* = 102), a primary cohort (*n* = 49), and 2 independent validation cohorts from 2 different hospitals (*n* = 49 and *n* = 45).	N/A
[35]	Epithelial ovarian cancer (EOC)	The study included a total of 334 patients with epithelial ovarian cancer and 101 patients with benign ovarian tumors. Among them, 168 patients with EOC and 51 patients with benign ovarian tumors are assigned to the training cohort, while 166 patients with EOC and 50 patients with benign ovarian tumors remained assigned to the test cohorts. The data were collected between 2010 and 2017.	N/A
[36]	Breast cancer	From November 2016 to March 2019, perfusion CT was carried out on 246 successive women who has been scheduled to receive treatment for invasive breast cancer. Of these, 241 cases were included in the study.	N/A
[37]	Bone cancer (osteosarcoma)	The researchers obtained an overall of 94 osteosarcoma expression data points for 1333 pseudogenes, along with corresponding clinical follow-up information, from the TARGET database.	TARGET
[11]	Lung cancer	This study used simulated data with different scenarios. It also used non-small cell lung cancer data (IPASS trial) to compare the efficacy of gefitinib versus carboplatin and paclitaxel. The phase III study involved 1217 patients who were randomized equally between the 2 treatment groups.	The details of the data are available in Supp
[38]	Gastric cancer	Clinicopathological data and expression profiles for lncRNAs in 350 patients diagnosed with gastric cancer obtained from the TCGA website.	TCGA
[39]	Prostate cancer	The research involved 19 patients with low- and intermediate-risk prostate cancer (characterized by Gleason-Score ≤ 7b, age ≤ 75 years, staging pT2, resection border R0) who underwent radical prostatectomy.	N/A
[40]	Five cancer types	Sequencing datasets and corresponding clinical data for several cancer types, including BLAC, BRCA, CESC, LAML, and LIHC, were obtained from Broad Institute GDAC Firehose.	GDAC
[41]	Several cancer types	Gene mRNA data, CNV data, DNA methylation data, SNP data, and clinical data for several cancer types, including PAAD, BRCA, KIRC, LGG, and STAD were obtained from the TCGA website.	TCGA
[42]	Renal cell cancer	H&E Slides and TCGA and ccRCC (ccRCC) cases from TCGA.	The details of the data are available in Supp

**Table 3:** Analysis of tree subgroup identification methods.

Tree Subgroup Identification Methods				
Method	Description	Objective Function	Limitations	Source Code
Interaction trees (**IT**) [52,53]	The algorithm adheres closely to the CART (classification and regression trees) method. It splits the data repeatedly by selecting the split that maximizes an objective function. The final tree is then trimmed using the Akaike information criterion.	Maximizing *p*-values	The variables in the smaller group may be considered predictive, although the exhaustive search for splits makes their determination uncertain. This is due to the fact that variables that provide more opportunities for splitting a node are more likely to be selected. Additionally, optimizing quantity results in biased estimates of treatment effects.	IT
Subgroup identification based on differential effect search (**SIDES**) [54]	The objective was to construct a set of subclasses by dividing a database into two subclasses at each parent group in a recursive manner so that the treatment effect is higher in one subcategory compared to the other. The data split remains until a pre-defined stopping rule is met. The approach resembles the IT method that incorporates treatment-split interactions into the splitting criteria. However, their approach only explores inside certain areas of the covariate space and creates various subgroups that may be of importance.	Locating multiple alternative subgroups by determining the *m* optimal splits of each node *t* that increase a *p*-value criterion	Amendments are made to the *p*-values based on heuristics to account for multiple divides and associations amongst the *p*-values. When a variable is chosen to split a node, it is not used for splitting any future nodes. Hence, SIDES cannot produce sub-groups of the structure {a <X≤b} with finite values of *a* and *b*.	SIDES
Virtual Twins (**VT**) [55]	This method employs RF to calculate the treatment effect of every examination, including split variables and their interactions. Categorical variables are transformed into 0–1 dummy variables. Next, CART is utilized to construct a classification or regression tree based on the projected variable values to identify the subgroups.	If a classification tree is applied, the two categories are determined by the estimated variable being above or below a pre-determined constant. If a regression tree is applied, the subgroups that have an estimated treatment effect greater than φ + 0.05 are considered as the final nodes.	The application of CART in VT enables the identification of subgroups as well as predictive variables, but the latter is prone to inaccuracies owing to the selection biases inherent to CART.	VT
Generalized unbiased interaction detection and estimation (**GUIDE**) [56,57,58]	GUIDE divides the data recursively to form a binary tree with its terminal nodes defining subgroups. The partitioning process continues until the number of samples in each node drops below a designated threshold. Afterwards, the CART cross-validation trimming technique is used to shrink the size of the tree. GUIDE is specifically developed to eliminate the issue of variable selection bias, which can compromise the accuracy of inferences from a tree structure. To mitigate bias, GUIDE employs a residuals chi-square analysis and bootstrap significance probabilities method for collaboration.	Minimizing residual sum of square using stepwise linear regression.	This method provides fast computational speed and is easily adaptable to different datasets. However, the interpretability of a tree structure decreases quickly as its complexity grows, making it harder to understand a tree with many splits compared to a traditional linear regression model.	GUIDE
Model-based recursive partitioning (**MOB**) [59,60]	MOB is a recursive partitioning method for automatically identifying patient subgroups based on predictive factors. A parametric model, such as a generalized linear model or a Weibull quicker failure time approach is fitted to the data at each node, with parameter values estimated by solving the score equations, which are partial derivatives of the log-likelihood. The variable used to divide a node is determined by examining the independence among each *X* variable and the scores related to the capture and therapy effect. If there are no significant test results, as determined by a predetermined level of significance with Bonferroni adjustments, the node is not divided. If a significant result is found, the variable with the smallest *p*-value is chosen, and the splitting end is chosen to minimize the total negative log-likelihood in the two sub nodes.	Minimizing *p*-values and minimizing the total negative log-likelihood in the two sub nodes.	MOB controls the error in variable selection at each node, ensuring that the chance of selecting a partitioning variable for splitting when all variables are actually independent of the scores is no higher than the nominal level. However, the use of multiple testing procedures can lead to reduced accuracy in detecting existing subgroups when there are many irrelevant partitioning variables.	MOB in supplemental materials.

**Table 4:** Analysis of non-tree subgroup identification methods.

Non-Tree Subgroup Identification Methods				
Method	Description	Objective Function	Limitations	Source Code
Finding heterogeneous treatment effects (**FindIt**) [61]	The main aim of their proposed method is to view the identification of heterogeneous therapy effects as a variable selection problem. A penalized SVM is introduced with two sparseness restrictions, one for the set of relevant therapy effect heterogeneity parameters and the other for the observed pre-therapy effect factors (two LASSO penalties) to find predictive biomarkers.	The authors formulated the support vector machine (SVM) as a corrected squared hinge-based loss objective function.	The results demonstrate that the FindIT method has low rates of false discoveries while maintaining strong discovery rates. However, this method has a drawback of being conservative, it performs worse for small sample sizes.	FindIt
Regularized outcome weighted subgroup identification (**ROWSi**) [62]	This technique is used to estimate a target function that directly shows the appropriate treatment for patients. Instead of modeling targets, the function uses patient outcomes weights. This allows the method to handle different types of outcomes, such as binary, continuous, time to event, and possibly contaminated results in a similar way. The initial step is to determine directional estimates from linear rules that define crucial patient subgroups. Afterwards, the comparative effects of treatments for these identified subgroups are estimated. A fused LASSO penalty has been used. A bootstrap method is used to construct confidence intervals.	The authors evaluated ROWSi’s performance by calculating sensitivity, specificity, prediction accuracy, and predicted outcome.	This approach is primarily utilized to identify the guidelines for assigning treatments. It is used to identify suitable patients for medication, unlike other subgroup methods which are typically utilized to find the appropriate medication for patients.	ROWSi in Supp material
Patient rule induction method (**PRIM**) [63]	A method to search for predictive signatures using the patient rule induction. The procedure includes the selection of an appropriate objective function for the search, the presentation of its process, and the description of a resampling technique to improve its performance. The performance of the procedure is evaluated through simulations and its practical applications are demonstrated on two real-world datasets in the field of oncology with a focus on survival responses.	A bump-hunting procedure is applied to one subset of the training sample to find subgroups, with the *p*-value of the treatment effect serving as the objective function. The other subset is then employed to select the final subgroup from the pool of candidates.	The objective function does not take into account the interaction effect condition.	PRIM
Sequential bootstrapping and aggregating of threshold from trees (**SeqBT**) [64]	A subgroup identification method for creating threshold multivariate biomarker signatures using resampled tree-based methods and variations of the Monte Carlo adaptive indexing method that incorporate variable selection.	Minimizing the *p*-value	Some drawbacks such as the constant threshold of defining the *p*-value can be adjusted and the need for regularization techniques to prevent overfitting.	SeqBT
Outcome weighted estimation (**OWE**) [65]	A typical context for identifying subgroups using weighting or A-learning in both randomized clinical trials and observational studies. It is based on the potential outcome approach of causal inference and uses the treatment variable *Z* that has two possible values (+1 and −1). The method employs a score function *f(X)* that minimizes a loss function *M (y, v*), which could be either squared error or logistic loss.	Squared error or logistic loss functions	There are some limitations such as, in order to determine the appropriate form of the function, a trade-off between bias and variance must be considered. When the sample size is large, cross-validation can be used to choose the best set of basis functions for a given dataset and loss function. If the number of covariates or basis functions is high, regularization techniques like lasso or elastic net can be used to prevent overfitting by selecting variables and stabilizing the model fitting process.	Personalized

## Data Availability

Not applicable.
